# Spatial Transcriptomics Analysis: Maternal Obesity Impairs Myogenic Cell Migration and Differentiation during Embryonic Limb Development

**DOI:** 10.3390/ijms25179488

**Published:** 2024-08-31

**Authors:** Yao Gao, Md Nazmul Hossain, Liang Zhao, Jeanene Marie Deavila, Nathan C. Law, Mei-Jun Zhu, Gordon K. Murdoch, Min Du

**Affiliations:** 1 Nutrigenomics and Growth Biology Laboratory, Department of Animal Sciences, Washington State University, Pullman, WA 99164, USA; yao.gao@wsu.edu (Y.G.); mdnazmul.hossain@wsu.edu (M.N.H.); deavilaj@wsu.edu (J.M.D.); nathan_law@wsu.edu (N.C.L.); gordon.murdoch@wsu.edu (G.K.M.); 2 College of Animal Science and Technology, Nanjing Agricultural University, Nanjing 210095, China; liang.zhao@njau.edu.cn; 3 School of Food Science, Washington State University, Pullman, WA 99164, USA; meijun.zhu@wsu.edu

**Keywords:** GeoMx spatial RNA sequencing, cell migration, embryonic myogenesis, maternal obesity, limb development

## Abstract

Limb muscle is responsible for physical activities and myogenic cell migration during embryogenesis is indispensable for limb muscle formation. Maternal obesity (MO) impairs prenatal skeletal muscle development, but the effects of MO on myogenic cell migration remain to be examined. C57BL/6 mice embryos were collected at E13.5. The GeoMx DSP platform was used to customize five regions along myogenic cell migration routes (myotome, dorsal/ventral limb, limb stroma, limb tip), and data were analyzed by GeomxTools 3.6.0. A total of 2224 genes were down-regulated in the MO group. The GO enrichment analysis showed that MO inhibited migration-related biological processes. The signaling pathways guiding myogenic migration such as hepatocyte growth factor signaling, fibroblast growth factor signaling, Wnt signaling and GTPase signaling were down-regulated in the MO E13.5 limb tip. Correspondingly, the expression levels of genes involved in myogenic cell migration, such as *Pax3*, *Gab1*, *Pxn*, *Tln2* and *Arpc*, were decreased in the MO group, especially in the dorsal and ventral sides of the limb. Additionally, myogenic differentiation-related genes were down-regulated in the MO limb. MO impedes myogenic cell migration and differentiation in the embryonic limb, providing an explanation for the impairment of fetal muscle development and offspring muscle function due to MO.

## 1. Introduction

The prevalence of obesity has been increasing for decades and is continuing to rise. It is estimated that one billion people will be obese by 2030, including one fifth of females [1,2]. Western-style food or high-fat diet (HFD) consumption is one of the major causes of obesity due to the excessive intake of energy and metabolic dysregulation [3]. Obesity not only adversely affects maternal health, but also impairs the metabolic health of offspring. Skeletal muscle serves as the largest metabolic organ [4]. The function of offspring’s skeletal muscle is impaired due to maternal obesity (MO), showing an altered muscle fiber composition [5] and suppressed exercise endurance and metabolic function [6]. These negative effects on the offspring’s skeletal muscle caused by MO have an embryonic origin [7], when the first wave of myogenic cells initiates. Our previous study showed that MO inhibits embryonic myogenesis at the single cell level [7]. The disruption of myogenesis at this stage results in lasting adverse effects on postnatal skeletal muscle growth [8,9].

Embryonic myogenesis is a spatiotemporal process that is governed by a group of myogenic regulatory factors: MYF5, MYOD, MYOG and MRF4 [10,11]. A portion of embryonic pluripotent stem cells within the somitic mesoderm develop into myogenic progenitor cells, characterized by the expression of the paired box transcription factors PAX3 and PAX7. These myogenic progenitor cells subsequently express MYF5 and transform into myoblasts, which eventually differentiate into myotubes [12]. Embryonic myogenesis initiates in the dermomyotome, where two lips (dorsomedial and ventrolateral) of the dermomyotome gradually mature into the myotome, a primitive muscle structure containing committed muscle cells [11]_._

However, for the development of the muscle fibers of limbs, diaphragm and tongue, myogenic cells need to be migrated from the dermomyotome. More precisely, the muscle in these locations comes from a group of myogenic cells with an extensive migratory capacity, which undergo epithelial-to-mesenchymal transition and delaminate from the ventrolateral lip of the dermomyotome [12]. In general, cell migration is vital for normal embryogenesis and morphogenesis. The directional migration of a cell includes four repeat steps: (1) the formation of protrusion; (2) adhesion; (3) contraction; and (4) retraction [13]. During embryonic myogenesis, the directional migration of skeletal myogenic progenitors is essential for the formation of limb muscle [14]. Myogenic cell migration is spatiotemporally regulated by a cascade of genes and signaling pathways independent of myogenesis.

Up to now, the effects of MO on myogenic cell migration during limb development remain undefined, primarily due to the small size of early embryos and the complexity of spatiotemporal regulation. The previously developed RNA in situ hybridization (ISH) technique has limitations regarding the localization of mRNA in embryonic tissues, including the limited number of targets analyzed concurrently and its low resolution, and its poor compatibility with formalin-fixed paraffin-embedded (FFPE) tissues [15]. The newly developed GeoMx™ RNA assay overcomes these technical defects and can detect multiplexed mRNA targets in FFPE tissues. In the GeoMx assay, the whole-transcriptome ISH probes are connected to indexing oligo barcodes with a photocleavable site. The GeoMx Digital Spatial Profiler (DSP) platform can cleave these barcodes and profile the transcripts in customized region-of-interests (ROIs) [16]. These characteristics enable us to reveal the spatial transcriptome changes during embryonic limb muscle development caused by MO. 

Taken together, this study aims to profile the spatial transcriptomes of embryonic limbs and decipher the mechanism of MO-induced developmental abnormalities in limb skeletal muscle, focusing on myogenic cell migration and differentiation.

## 2. Results

### 2.1. Maternal HFD Feeding Alters Spatial Transcriptome of E13.5 Embryonic Limb

**Figure 1:**
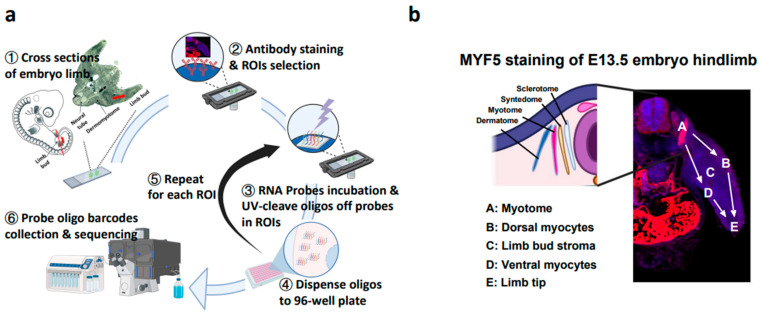
Schematic diagrams of GeoMx DSP and experiment design. (**a**) Workflow of GeoMx DSP platform. (**b**) Schematic diagram (**left**) and immunohistochemical staining (**right**) of the cross-section of E13.5 embryonic hind limb. Red fluorescence represents the MYF5 staining. ROI, region of interests.

Using the GeoMx Digital Spatial Profiler (DSP), spatially resolved gene expression data from embryonic limbs were captured (Figure 1a). Cross-sections of fixed embryo hind limbs were stained with barcoded ISH oligos probes that bind to endogenous mRNA transcripts. In this study, five ROIs along the migration route (A → E), annotated as A (myotome), B (dorsal limb), C (limb bud stroma), D (Ventral limb) and E (limb tip), were selected based on the limb morphology and MYF5 staining (Figure 1b). The GeoMx probes were then photo-cleaved and the expression barcodes of each ROI were collected separately and used for downstream sequencing and data processing.

**Figure 2:**
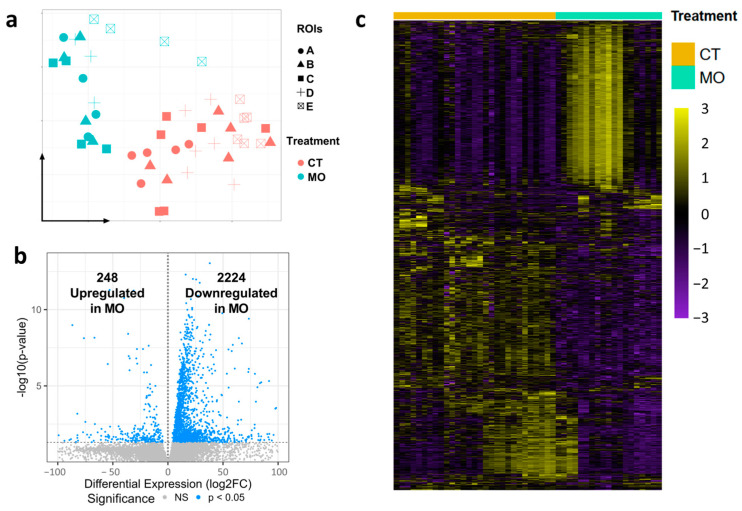
Maternal HFD feeding alters spatial transcriptomes of E13.5 embryonic limb. (**a**) UMAP plot of principal component analysis (PCA) based on treatments (CT and MO) and segments (ROIs). (**b**) Volcano plot of DEGs upregulated and downregulated in MO group compared with CT group. (**c**) The gene expression heatmap of CT and MO groups. CT, control; MO, maternal obesity; DEG, differentially expressed genes; A, myotome; B, dorsal limb; C, limb bud stroma; D, Ventral limb; E, limb tip; UMAP, Uniform Manifold Approximation and Projection.

After data filtering and processing, uniform manifold approximation and projection (UMAP) plots were used to spatially define the data among different ROIs (A–E) and treatments (CT and MO), showing a different expression profile and a clear separation between CT and MO samples (Figure 2a). The volcano plot showed that 2224 protein-coding genes were down-regulated, and that 248 protein-coding genes were up-regulated in the MO group compared to the CT group in the E13.5 limb (Figure 2b). The gene expression heatmap demonstrated the significant alteration of transcriptomes between the CT and MO groups (Figure 2c). 

### 2.2. Maternal HFD Feeding Inhibits Myogenesis and Myogenic Cell Migration in E13.5 Embryonic Limb

**Figure 3:**
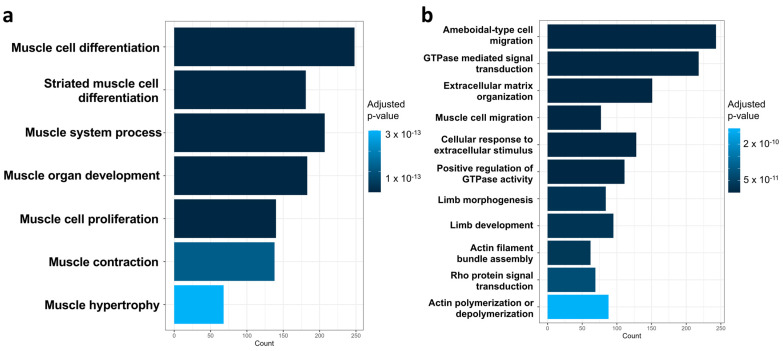
GO enrichment analysis of downregulated biological processes in MO compared with CT group. (**a**). Down-regulated myogenesis-related GO biological process terms in MO compared with CT group. (**b**). Down-regulated migration-related GO biological process terms in MO compared with CT group. CT, control; MO, maternal obesity; GO, gene ontology.

Regardless of ROIs, the gene ontology (GO) enrichment analysis displayed an overall down-regulation of biological processes including muscle cell differentiation, muscle system process, muscle organ development, muscle cell proliferation, muscle contraction and muscle hypertrophy (Figure 3a). In addition, the migration-related biological processes were down-regulated in MO compared with the CT group, such as ameboidal-type cell migration, muscle cell migration, extracellular matrix organization and limb morphogenesis/development. Consistently, the regulatory signaling pathways involved in cell migration such as GTPase signal transduction, Rho signal transduction and the cellular response to extracellular stimulus were also down-regulated in the E13.5 limbs of MO compared with the CT group. In support, actin filament polymerization, contraction and assembly were down-regulated in the MO group (Figure 3b).

Taken together, the integrated transcriptomic data regardless of ROIs showed suppressed myogenic differentiation and myogenic cell migration in MO E13.5 embryo limbs. 

### 2.3. MO Suppresses Migration Signal Factors Released from the E13.5 Limb Tip 

**Figure 4:**
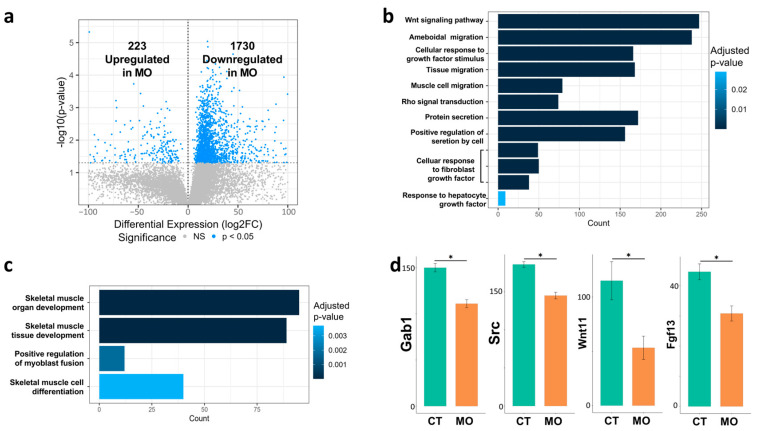
Spatial transcriptome analysis of E13.5 limb tip (ROI_A). (**a**) Volcano plots of DEGs up-regulated and down-regulated in the MO embryonic limb tip compared with those in the CT group. (**b**) Down-regulated migration-related GO biological process terms in the limb tip of the MO embryo compared with the CT group. (**c**). Down-regulated myogenesis-related GO biological process terms in the limb tip of the MO embryo compared with the CT group. (**d**). Expression level of genes involved in HGF, Wnt and FGF signaling in the E13.5 limb tip. Asterisk (*) indicated a significant difference (*p* < 0.05). CT, control; MO, maternal obesity; ROI, region-of-interests; GO, gene ontology.

To further investigate the spatial transcripts, we focused on ROI_E (limb tip), where cells release growth factors and chemo-attractants to guide the migration of myogenic cells from ROI_A (myotome). The volcano plot showed that about 1730 genes were down-regulated and 223 genes were up-regulated in the E13.5 limb tip of MO compared to the CT treatment (Figure 4a). The GO enrichment analysis showed that several signaling factors such as Wnt, FGF and HGF were repressed in the MO E13.5 limb tip. The muscle cell migration and Rho signaling were also down-regulated (Figure 4b). In addition, myogenesis-related GO biological processes such as skeletal muscle organ/tissue development, myoblast fusion and differentiation were suppressed in the E13.5 limb tip in MO embryos (Figure 4c). We further compared the expression levels of *Gab1*, *Src*, *Wnt11* and *Fgf13* genes, which were all down-regulated in the MO limb tip. In summary, MO inhibited the release of migration signaling factors in the E13.5 limb tip. 

### 2.4. Integrated Analysis of Transcriptomes Demonstrates the Suppression of Cell Migration and Myogenesis in the MO E13.5 Limb 

**Figure 5:**
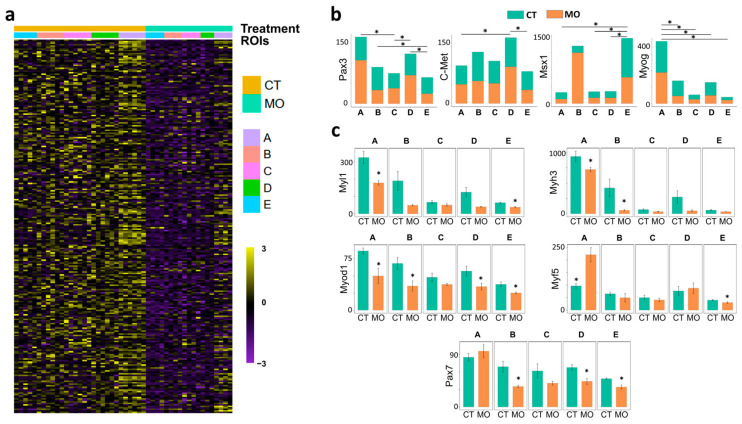
Maternal obesity inhibits the expression of genes involved in myogenesis in the E13.5 embryonic limb. (**a**) The expression heatmap of myogenesis-related genes between CT and MO treatments. (**b**) Comparison of gene expression among different ROIs. (**c**). The myogenic gene expression between CT and MO treatments among different ROIs. Asterisk (*) indicates significant difference (*p* < 0.05). CT, control; MO, maternal obesity; ROI, region-of-interests; A, myotome; B, dorsal limb; C, limb bud stroma; D, Ventral limb; E, limb tip.

**Figure 6:**
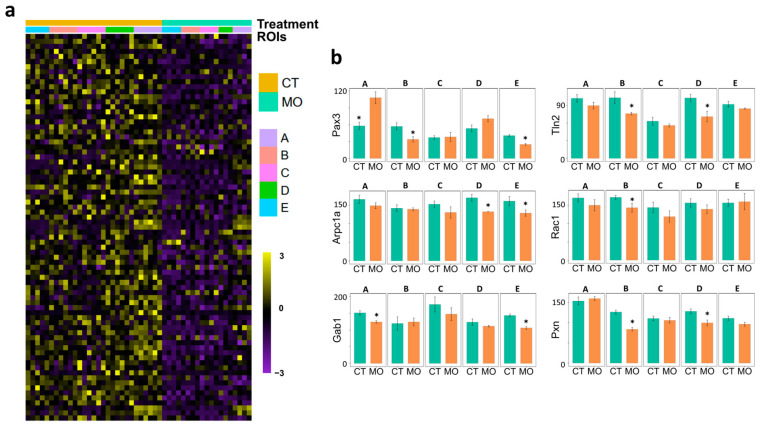
Spatial expression of selected genes between treatments. (**a**) The expression heatmap of cell migration-related genes between CT and MO treatments. (**b**). The migration-related gene expression between CT and MO treatments at different ROIs. Asterisk (*) indicates significant difference (*p* < 0.05). CT, control; MO, maternal obesity; ROI, region-of-interests; A, myotome; B, dorsal limb; C, limb bud stroma; D, Ventral limb; E, limb tip.

To explore the alteration caused by MO in detail, we analyzed the transcriptomic changes in ROIs along the migration path and specifically targeted the genes related to myogenesis and cell migration. Regarding myogenesis, the heatmap clearly showed MO decreased myogenic-related genes (Figure 5a) and migration-related genes (Figure 6a). There were several genes that could reflect the migration routes of myogenic cells from ROI_A (myotome) → B (dorsal limb) → E (limb tip) or from ROI_A (myotome) → D (ventral limb) → E (limb tip). For example, *Pax3* (marker of migrating pre-muscle cell), *c-Met* (HGF receptor) and *Myog* (terminal myogenic factor) displayed significantly higher expression in ROI_B and D, while the pro-migration factor *Msx1* showed a gradual increase from ROI_A to E (Figure 5b).

Because cell migration and myogenesis are orchestrated by many regulatory genes, we further analyzed the expression of the most important genes involved in migration and myogenesis. Myogenic factors such as *Pax7* (ROI_B, D and E), *Myod1* (ROI_A, B, D and E), *Myf5* (ROI_A and E), *Myl1* (ROI_A and E) and *Myh3* (ROI_A and B) were inhibited in corresponding ROIs of the MO limb (Figure 5c). Finally, the expression levels of *Pax3* (ROI_A, B and E), *Gab1* (ROI_A and E) and *Rac1* (ROI_B), which are involved in myogenic cell migration, were decreased in corresponding ROIs of the MO limb. The expression levels of *Pxn* (ROI_B and D), *Tln2* (ROI_B and D), and *Arpc1a* (ROI_D and E), which play important roles in actin filament assembly and contraction, were decreased in corresponding ROIs of the MO limb (Figure 6b). 

Overall, ROI_C (Limb bud stroma) was the region least affected by MO. By contrast, ROI_A, B, D and E, the regions that myogenic cells migrate to and reside in, were significantly impacted by MO.

## 3. Discussion

MO impairs fetal skeletal muscle development, a detrimental consequence persisting throughout the postnatal lifespan [17]. Our lab recently revealed the single-cell transcriptome changes that occur in the whole embryo due to MO and found that MO repressed myogenesis at the early E9.5 embryonic stage [7,18]. However, the negative effects of MO on limb muscle development remain undefined due to technical challenges. Mouse limb morphogenesis initiates around E9.0, and structural elements such as tendons, skeletal muscle and vessels gradually form during E12.5 to E15.5 [19]. During this period, myogenic cells in the myotome migrate along the limb axis (Figure 1b). In this study, we found that MO suppressed the migratory capability of myogenic cells in the E13.5 embryos. 

We found that myogenesis and cell migration were repressed in MO ROIs, except for the ROI_C (limb bud stroma). The ROI_C served as a negative control because it mainly develops into limb bones that no myogenic cells reside in [20]. Our data showed similar muscle migration routes (Figure 1b) and the lower expression levels of *Pax3* and *Myog*, two myogenic factors [21], in the ROIs of MO, showing the suppression of myogenesis. The limb tip eventually differentiates into hand/feet [22], while the dorsal and ventral limb muscle mass gives rise to major skeletal muscle along the arm/leg [23]. Accordingly, the ROI_E (limb bud) showed a lower expression level of myogenic markers such as *Myh3* [24] and *Myl1* [7] compared to ROI_B (dorsal-) and D (ventral-muscle mass) (Figure 5c). The ROI_A (myotome) displayed the highest expression level of myogenic genes, which is expected because the myotome is the origin of myogenic cells and appears earlier than the limb muscle [14]. 

Cell migration is regulated collaboratively by intracellular and extracellular pathways. Extracellular signals including chemo-attractants and growth factors are usually secreted by nonmigrating cells along the migration routes and function as spatial guides [25]. In the limb, the mesenchymal cells in ROI_E (limb tip) release hepatocyte growth factor (HGF), which attracts the migrating myogenic cells via HGF/MET signaling [26]. Our ROI_E-specific analysis showed that MO inhibited the HGF signaling (Figure 4d) in this location. In addition, Wnt and FGF signaling were also attenuated in the MO limb tip, indicating the decreased migratory signals for myogenic cells [27,28,29]. Correspondingly, the genes involved in the HGF (*Gab1*, *Src*) [30,31], Wnt (*Wnt11*) [27] and FGF (*Fgfgr13*) [32] signaling pathways were down-regulated in MO limbs (Figure 4d). One of the most important GTPase family proteins involved in migratory regulation is Rac1. After activation, Rac1 promotes actin filament nucleation and polymerization via the activation of the ARP2/3 complex [14]. The re-arrangement of actin filament is essential for cell migration [33]. The monomeric G-actins polymerize and assemble to form filamental F-actins (actin filaments), a dynamic process. When actin filaments grow at the plus end but shrink at their minus end, the filament behaves like a treadmill to propel cell migration. ARP2/3 helps to induce the nucleation of actin filament [34]. Consistently, our data showed that the expression levels of *Rac1* and *Arpc1a* (subunits of ARP2/3 complex [35,36]) decreased in the MO limb along the migration routes (ROI_A to ROI_E) (Figure 6b).

Cell migration requires the anterior portion of cells to protrude and attach onto the substrate surface, which is mediated by proteins that form focal adhesions, including integrin [37], focal adhesion kinase (FAK, also known as PTK2) [38], paxillin (PXN) [39], talin (TLN) [40], and vinculin (VCL) [41]. These proteins function as the structural proteins that link the extracellular matrix to the intracellular actin filaments [42]. In addition, cells reshape themselves by releasing matrix metalloproteinases to remodel the extracellular matrix along the migration route [43]. The expression levels of genes such as *Pxn* and *Tln2* (Figure 6b) and the capacity for extracellular matrix organization (Figure 3b) were decreased in the MO limb, indicating a repressed migratory capacity.

## 4. Conclusions

**Figure 7:**
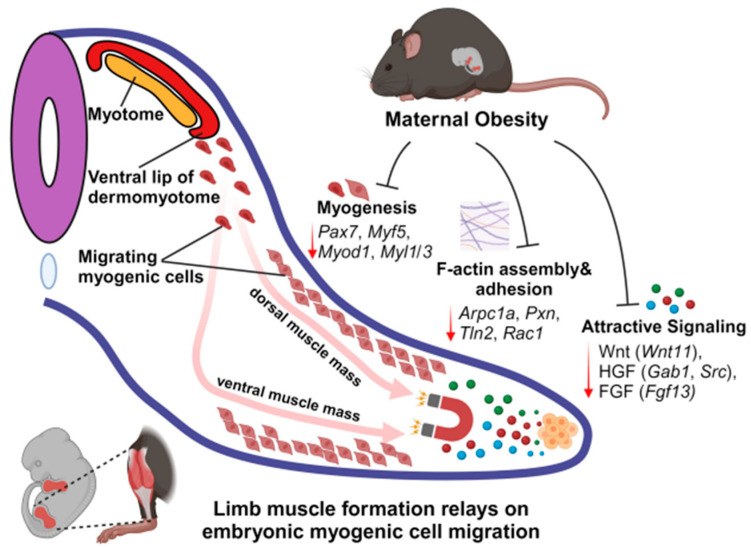
Schematic diagram showing inhibition of myogenic cell migration due to maternal obesity. The limb muscle formation relies on embryonic myogenic cells migrating along the myotome to the limb axis. MO inhibited the expression of genes involved in myogenesis and migration. The genes involved in myogenesis such as *Pax7*, *Myf5*, *Myod1* and *Myl1/3* and genes involved in F-actin assembly and adhesion such as *Arpc1a*, *Pxn*, *Tln2* and *Rac1* were decreased in the MO limb, reducing the cell migratory capacity. Genes involved in migration attractive signaling such as *Wnt11*, *Gab1*, *Src* and *Fgf13* were decreased, disturbing cell migration.

We profiled the spatial transcriptomes of the E13.5 limb using the GeoMx DSP platform and precisely described the negative effects of MO on limb myogenic cell migration and differentiation, showing the suppression of both myogenic cell migration and differentiation due to MO (Figure 7). These data provide valuable transcriptional information for the further exploration of underlying mechanisms and the discovery of molecular targets for novel therapeutics, in order to improve the embryonic myogenesis and health of MO offspring born to the increasing number of obese mothers.

## 5. Materials and Methods

### 5.1. Animal Handling and Sample Collection

Female C57BL/6J mice (8 weeks old; 000664, The Jackson Lab, Bar Harbor, Maine) were randomly assigned to high-fat diet (MO) and control (CT) groups per our established protocol [7]. Briefly, the control diet (10% energy from fat, D12450H, Research Diets, New Brunswick, NJ, USA) and high-fat diet (45% energy from fat, D12451) were fed ad libitum to mice in the CT and MO groups, respectively. After 2 to 3 months of feeding, obesity was induced in the MO group, indicated by a 20% greater average body weight than that of CT mice. Next, C57BL/6J male mice (4 months old) fed with a regular chow were used to mate with the experimental females. Successful mating was confirmed by the presence of vaginal plug and designated as E0.5 (0.5 embryo day after fertilization). After mating, pregnant mice were continuously fed with their respective diets until embryo collection at E13.5. All animal studies were conducted in AAALAC-approved facilities and approved by the Institutional Animal Use and Care Committee at Washington State University (Permit No. 6712.04).

Pregnant mice were anesthetized by carbon dioxide inhalation and euthanized by cervical dislocation at E13.5. The embryos were dissected and the caudal portions (~27 to 55 somite pairs) [44] containing hind limbs (Figure 1a) were separated and fixed in formalin for 12 h, then stored in 70% ethanol at 4 °C for later processing. 

### 5.2. Embryo Collection and Spatial RNA Sequencing

To avoid RNA degradation, the whole process was conducted under RNase-free conditions. All equipment was treated using RNase Away decontaminant (#10328011, Thermo Fisher, Waltham, MA, USA). All water used in this study was RNase-free water (#A57775, Thermo Fisher). Samples were processed per the standard protocol for formalin-fixed paraffin-embedded tissue (FFPE) preparation [45], which includes fixation (10% formalin), dehydration (ethanol series from 70% to 100%), substitution (100% xylene), paraffin infiltration and embedding. 

The paraffin-embedded samples were then sectioned at a thickness of 5 µm and mounted on poly-L-lysine-coated glass slides. The further preparation of slides strictly followed the manual provided by GeoMx^®^ DSP, Nanostring [46]. In brief, 5 μm FFPE sections were dewaxed, target retrieved, digested with proteinase K, post-fixed, and then incubated overnight with GeoMx RNA detection probes. Stringent washes were performed, followed by the addition of fluorescently labeled antibodies for localizing regions-of-interest (ROIs). User-defined ROIs were then profiled through region-specific cleaving to collect the photocleaved indexing oligos in the GeoMx Digital Spatial Profiler (Nanostring, Seattle, WA, USA). A total of 6 cross sections from CT embryos (*n* = 6) and 6 cross sections from HFD embryos (*n* = 6) were selected. Each embryo randomly selected from one pregnant mouse was regarded as one biological replicate. Based on the MYF5-positive staining and the morphologic location, 5 ROIs on each section were manually selected to construct the transcriptomic library. A total of 29 libraries from CT embryo ROIs and 19 libraries from HFD embryo ROIs were successfully established. The morphologic markers used in this study include Syto13 nucleic acid stain (included in the NanoString Morphology Marker Kits, Seattle, WA, USA) and customized MYF5 fluorescent antibody (PA5-47565, Thermo Fisher). Cleaved indices were sequenced in an Illumina next seq 2K (San Diego, CA, USA) and quantified to generate a digital quantification of the RNA expression within a spatial context.

### 5.3. Sequencing Data Analysis

The raw sequencing data were assembled and transferred into a .DCC file (digital conversation counts) on the Illumina Base Space analysis platform. The pre-processed .DCC data file was integrated into R and analyzed following the GeoMx Workflows (v 3.6.0) Vignette [47]. Briefly, the GeoMx Mouse Whole Transcriptome Atlas (Mm_R_NGS_WTA_v1.0.pkc) was used to configurate the raw data. The data were filtered (minSegmentReads = 10, percentTrimmed = 80, percentStitched = 80, percentAligned = 80, percentSaturation = 10, minNegativeCount = 1, maxNTCCount = 9000, minNuclei = 10, minArea = 100) and normalized (norm_method = “quant”) before generating the uniform manifold approximation and projection (UMAP) graph. After filtering, a total of 12,053 genes were identified. An analysis of differentially expressed genes (DEGs) was conducted to find the DEG profile of the CT and HFD groups (mixedModelDE). The significance was confirmed using the linear mixed-effect model with *p* value < 0.05. The Gene Ontology (GO) analysis was performed using R package, cluster Pro-filer v3.18.0. All data were visualized and analyzed by Prism GraphPad (V.7.0, San Diego, CA, USA) and R package ggplot2 (v3.0.0), presented as mean ± SD.

## Data Availability

The raw data has been deposited in the database: https://doi.org/10.5281/zenodo.13140203 (accessed on 31 July 2024). The R code is available in https://github.com/yaogao1994/GeoMx_DSP_RNA_seq (accessed on 31 July 2024).
